# Activation of Multiple Apoptotic Pathways in Human Nasopharyngeal Carcinoma Cells by the Prenylated Isoflavone, Osajin

**DOI:** 10.1371/journal.pone.0018308

**Published:** 2011-04-12

**Authors:** Tsung-Teng Huang, Fu-Guo Liu, Chia-Fong Wei, Chia-Chen Lu, Chang-Chieh Chen, Hung-Chi Lin, David M. Ojcius, Hsin-Chih Lai

**Affiliations:** 1 Department of Life Sciences, National Central University, Taoyuan, Taiwan, Republic of China; 2 Department of Medical Biotechnology and Laboratory Sciences, Chang Gung University, Taoyuan, Taiwan, Republic of China; 3 Department of Respiratory Therapy, Fu Jen Catholic University, Taipei, Taiwan, Republic of China; 4 Health Sciences Research Institute and School of Natural Sciences, University of California Merced, Merced, California, United States of America; 5 Center for Molecular and Clinical Immunology, Chang Gung University, Taoyuan, Taiwan, Republic of China; University Paris Sud, France

## Abstract

Osajin is a prenylated isoflavone showing antitumor activity in different tumor cell lines. The underlying mechanism of osajin-induced cancer cell death is not clearly understood. In the present study, the mechanisms of osajin-induced cell death of human nasopharyngeal carcinoma (NPC) cells were explored. Osajin was found to significantly induce apoptosis of NPC cells in a dose- and time-dependent manner. Multiple molecular effects were observed during osajin treatment including a significant loss of mitochondrial transmembrane potential, release of cytochrome *c* into the cytosol, enhanced expression of Fas ligand (FasL), suppression of glucose-regulated protein 78 kDa (GRP78), and activation of caspases-9, -8, -4 and -3. In addition, up-regulation of proapoptotic Bax protein and down-regulation of antiapoptotic Bcl-2 protein were also observed. Taken together, osajin induces apoptosis in human NPC cells through multiple apoptotic pathways, including the extrinsic death receptor pathway, and intrinsic pathways relying on mitochondria and endoplasmic reticulum stress. Thus, osajin could be developed as a new effective and chemopreventive compound for human NPC.

## Introduction

The flavonoids are a heterogeneous group of phenolic compounds showing diverse biological effects such as antioxidant, antiviral, anticancer, anti-inflammatory, and antiallergic activities [1], [2]. Flavonoids are frequently used in oncology to reduce the side effects of cytostatics and enhance the therapeutic effects [3], [4].

Osajin is a flavonoid compound isolated from the fruit of *Maclura pomifera*, a tree belonging to the Moraceae or mulberry family, and is commonly referred to as “osajin orange, hedge apple, bow wood, and horse apple” [5], [6]. Through suppression of oxidative stress, osajin is cardioprotective by attenuating the myocardial dysfunction provoked by ischemia-reperfusion in rat hearts [7]. In addition, osajin exhibits growth inhibitory activity on six human cancer cell lines, including kidney, lung, prostate, breast, melanoma and colon cancer cells [8]. In contrast, the low toxicity of osajin against hepatocytes was also reported [8]. Despite the increasing therapeutic interest in osajin, the mechanisms of action of osajin on nasopharyngeal carcinoma (NPC) cells remain unaddressed.

NPC is the most common type of head-and-neck cancer worldwide, with a distinct racial and geographic distribution across the world [9]. It is endemic in Southeast Asia, Southern parts of China, the Mediterranean basin, and the Middle East [10]. Etiologic factors identified for NPC include genetic susceptibility, Epstein-Barr virus (EBV) infection, environmental factors (including chemical carcinogens) and dietary factors [11]. NPC shows relatively high sensitivity to radiation, and radiotherapy remains the mainstay treatment for early stages of NPC. Moreover, some pathological types (types 2 and 3) are chemosensitive at all stages of the disease [12], [13]. The concurrent combination of radiotherapy and chemotherapy has improved the outcome for patients with advanced stages of NPC. However, side effects such as fatal toxicity and the poor outcome of recurrent disease with chemotherapy were observed in many patients, indicating that the development of more effective and less toxic alternatives is urgently needed.

Dysregulation of apoptosis or programmed cell death is emerging as a key factor for cancer development [14]. Accumulating evidence clearly indicates that apoptosis is a crucial mechanism used by dietary bioactive agents for chemoprevention of cancers [15]. Apoptosis is characterized by morphologic and biochemical changes including cell shrinkage, chromatin condensation, and DNA fragmentation [16]. At the molecular level, apoptosis is tightly regulated by the activation of the aspartate-specific cysteine proteases known as caspases [17], and both extrinsic and intrinsic pathways have been described. The extrinsic, death receptor-mediated pathway is activated at the cell surface when a specific ligand binds to its corresponding cell-surface death receptor. Stimulation of death receptors of the tumor necrosis factor (TNF) receptor superfamily such as Fas (APO-1/CD95) results in receptor aggregation and recruitment of the adaptor molecule, Fas-associated death domain (FADD). Upon recruitment, caspase-8 becomes activated and initiates apoptosis by direct cleavage of downstream effector caspases, most notably caspase-3 [18]. Activation of caspase-8 can also result in cleavage of Bid, a Bcl-2 family protein with a BH3 domain only, which in turn translocates to the mitochondria to release cytochrome *c*, thereby initiating a mitochondrial amplification loop [19]. In the mitochondria-mediated pathway, activation of mitochondria is accompanied by the release of cytochrome *c* from mitochondria, which amplify apoptosis in both the extrinsic and intrinsic pathways. The cytochrome *c* then interacts with apoptosis protease-activating factor-1 (Apaf-1), ATP and procaspase-9 to form a supramolecular complex called the apoptosome. The apoptosome, in turn, activates caspase-9 through autocatalysis, and the latter then activates caspase-3, resulting in apoptosis [20]. Furthermore, the mitochondria-dependent apoptotic pathway is tightly regulated by Bcl-2 family proteins such as Bax and Bak. Both are proapoptotic members activated by a variety of apoptotic stimuli, leading to oligomerization and insertion into the mitochondrial outer membrane to release cytochrome *c* [21]. The intrinsic pathway is initiated within the cell when intracellular stress acts via BH3-only proteins such as Bid and leads to activation of Bax and Bak. This results in apoptosis independently of the surface-bound receptors such as Fas.

Recent studies have revealed that a third subcellular compartment, the endoplasmic reticulum (ER), is implicated in apoptosis induced by ER stress [22], [23]. ER stress activates the unfolded protein response (UPR) and the ER-resident cysteine protease, caspase-12, leading to caspase-3 activation and apoptosis [24]. However, although murine caspase-12 is an active enzyme, the human homolog, caspase-12, contains several mutations that render it non-functional [25]. In contrast, human caspase-4, which is also a resident of the ER is the counterpart of murine caspase-12 and is activated by ER stress [26]. Induction of glucose-regulated protein GRP78, also referred to as BiP (immunoglobulin heavy-chain binding protein), has been widely used as a marker for ER stress and the onset of UPR. Due to its antiapoptotic properties, stress induction of GRP78 represents an important prosurvival component of the evolutionarily-conserved UPR. Recent evidence shows that the microenvironment of tumors can stimulate physiological ER stress, and GRP78 is up-regulated in many types of cancer cells lines and tumor biopsies [27]. The ER stress-induced apoptosis modulator also includes CCAAT/enhancer-binding protein (C/EBP)-homologous protein (CHOP)/growth arrest and DNA-damage-inducible gene 153 (GADD153). Overexpression of CHOP plays a central role in apoptosis [28], including the dephosphorylation of the proapoptotic BH3-only protein Bad [29] and down-regulation of Bcl-2 expression [30].

In the present study, the ability of osajin to kill NPC cell lines was characterized. Osajin was found to reduce cell viability of NPC cells through apoptosis. The underlying mechanism was found to be due to activation of capases-9, -8, -4 and -3. Disruption of the mitochondrial membrane potential, release of cytochrome *c* from mitochondria, up-regulation of FasL and Bax, and down-regulation of GRP78 and Bcl-2 were also observed. Due to its broad apoptotic effects on NPC cells, osajin should be further explored for its therapeutic potential against NPC.

## Results

### Osajin reduces the viability of human NPC cells

**Figure 1:**
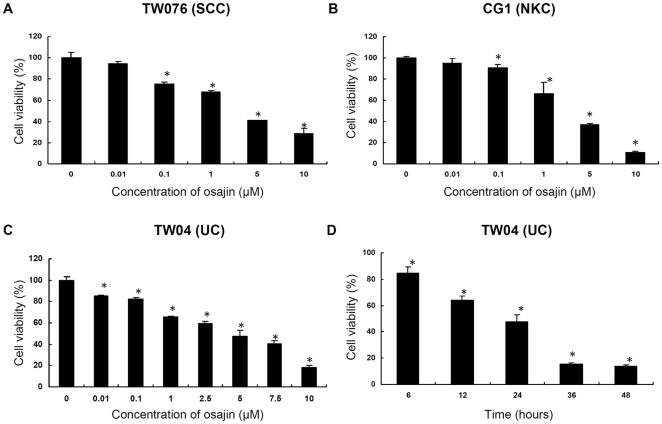
Effect of osajin on the viability of human NPC cells. (**A**) TW076 cells, (**B**) CG1 cells, and (**C**) TW04 cells were treated with various concentrations (0.01–10 µM) of osajin for 24 h. (**D**) TW04 cells were treated with 5 µM osajin for the indicated times. Cell viability was determined using the MTT assay. The data are presented as the means ± SE of three individual experiments (*P<0.05 versus 0 µM control).

The effects of osajin on the viability of human NPC cells were first studied. Three different types of NPC cell lines were treated with increasing concentrations of osajin for 24 h, and were followed by the MTT assay. As shown in Figure 1A, B and C, osajin significantly decreased the viability of TW076, CG1 and TW04 cells in a dose-dependent manner. In addition, a time-dependent inhibition of the viability of TW04 cells was also observed (Figure 1D). However, osajin at a concentration of 10 µM did not show significant effect on the viability of the human bronchial epithelial cell line BEAS-2B (data not shown). Thus, osajin treatment reduced the cell viability of different histological types of NPC cell lines.

### Osajin induces apoptosis and DNA fragmentation in TW04 cells

**Figure 2:**
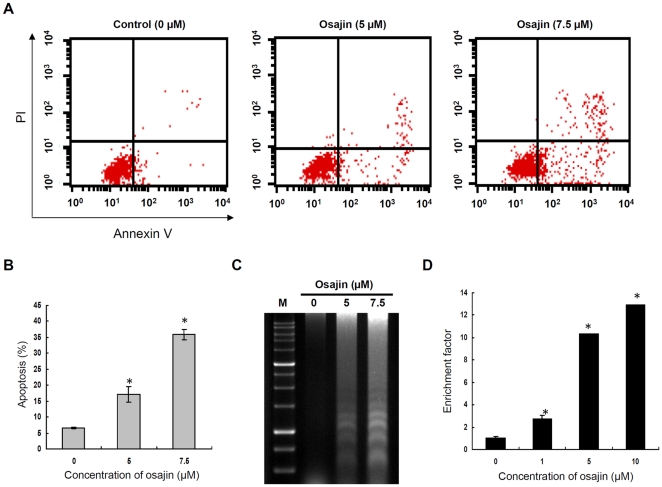
Osajin treatment induces apoptosis and DNA fragmentation in TW04 cells. Cells were treated with 0, 5 or 7.5 µM osajin for 24 h and stained with Annexin V-FITC and PI. (**A**) Cells in the upper-right (UR) portion represent cells undergoing late apoptosis, whereas cells in the lower-left (LL) and lower-right (LR) portions are viable and early-apoptotic cells, respectively. (**B**) The data indicate the percentage of annexin V-positive cells (apoptosis). (**C**) Agarose gel electrophoresis of DNA fragments from cells treated with various concentrations of osajin for 24 h. M: 1 kb DNA ladder size marker. (**D**) Cells were treated with various concentrations of osajin for 24 h in a 96-well plate, and the enrichment of nucleosomes in the cytoplasm was determined using the Cell Death Detection ELISA^PLUS^ kit. Results are shown as the means ± SE of four independent experiments (*P<0.05 versus 0 µM control).

As type 3 NPC comprises over 95% of NPC in high-incidence areas [9], the undifferentiated carcinoma cell line TW04 (type 3 NPC) was used for subsequent mechanistic studies. To determine whether the cytotoxic effect of osajin was mediated via apoptosis, annexin V-FITC/PI double staining was performed. As shown in Figure 2A and B, the percentage of apoptotic TW04 cells increased from 6.6% in control cells to 17% and 36% after treatment with 5 and 7.5 µM osajin, respectively.

A prominent feature of apoptosis is the degradation of chromatin DNA at internucleosomal linkages. The ability of osajin to induce DNA fragmentation in NPC cells was therefore addressed. For this purpose, TW04 cells were treated with 5 and 7.5 µM osajin for 24 h and DNA was fractionated by agarose gel electrophoresis. A typical ladder pattern of internucleosomal fragmentation was observed in cells treated with osajin. The ladder pattern was absent in untreated cells (Figure 2C). A Cell Death Detection ELISA kit that specifically detects cytoplasmic histone-associated DNA fragments was also used. Compared with untreated control cells, osajin treatment of TW04 cells resulted in significant dose-dependent apoptosis, with a 10 to 13-fold increase in internucleosomal DNA fragmentation by treatment with 5 and 10 µM osajin, respectively (Figure 2D).

### Osajin induces caspase-mediated apoptosis in TW04 cells

**Figure 3:**
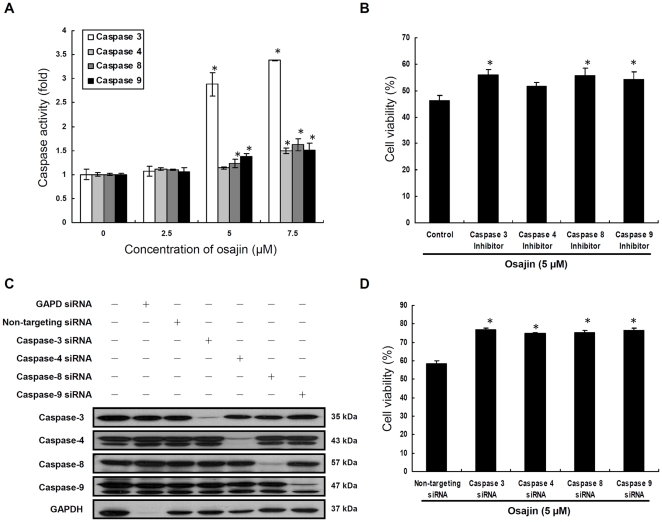
Osajin induces caspase-dependent apoptosis in TW04 cells. (**A**) Cells were treated with various concentrations of osajin for 24 h. The cytosolic fraction of the cells was then analyzed for the activities of caspases-3, -4, -8 and -9 using caspase colorimetric assay kits (*P<0.05 versus 0 µM control). (**B**) Cells were treated with 5 µM osajin for 24 h in the presence of caspase-3, -4, -8 or -9 inhibitors, and then cell viability was determined by the MTT assay. The data shown are the means ± SE of three individual experiments (*P<0.05 versus control cells treated with osajin alone). (**C**) Cells were transfected with caspase-3, -4, -8, -9, GAPD control or non-targeting control siRNA (25 nM) for 48 h, and protein extracts were prepared for Western blot using caspase-3, -4, -8, -9 and GAPDH antibodies. (**D**) Cells were transfected with the indicated siRNA, and cell viability after 5 µM osajin treatment for 24 h was estimated by the MTT assay. Results were expressed as the means ± SE of three individual experiments (*P<0.05 versus non-targeting control siRNA-transfected cells).

Apoptosis is executed by a family of cysteine-dependent aspartate-specific proteases known as caspases. As caspase-3 is a key protease associated with DNA fragmentation, we investigated the activity of caspase-3 in osajin-treated TW04 cells by the colorimetric assay. The enzyme activity of caspase-3 stimulated by osajin was measured using a specific substrate, DEVD-*p*NA. Caspase-3 activity was markedly increased by osajin treatment. A 1.07, 2.88 and 3.38-fold increase in DEVD-*p*NA cleavage was observed after incubation with 2.5, 5 and 7.5 µM osajin for 24 h, respectively, compared with untreated controls (Figure 3A).

To further elucidate whether activation of caspase-3 by osajin involves extrinsic (death receptor), intrinsic (mitochondrial) or ER stress-mediated apoptotic pathways, the activities of caspase-9, -8 and -4 , which are the apical proteases in the intrinsic, extrinsic, and ER-mediated pathway, respectively, were also analyzed by the colorimetric assay in TW04 cells. As shown in Figure 3A, osajin increased the activity of capase-8 and -9 in a dose-dependent manner compared with untreated cells. A 1.1 to 1.62-fold increase for caspase-8 activity and 1.06 to 1.51-fold increase for caspase-9 activity were observed after incubation with 2.5–7.5 µM osajin for 24 h. By contrast, while caspase-4 activity remained unaffected by 2.5 and 5 µM osajin treatments, the activity was significantly increased up to 1.51-fold by 7.5 µM osajin compared with untreated cells. Further treatment of TW04 cells with 10 µM caspase-3, -8 or -9 inhibitors before 5 µM osajin challenge resulted in significant increase of cell viability (Figure 3B). However, treatment with 10 µM caspase-4 inhibitor caused a smaller increase in cell viability of osajin-treated TW04 cells (Figure 3B).

To further confirm the role of caspase-3, -4, -8 and -9 in osajin-induced apoptosis, we performed gene knockdown studies using caspase-3, -4, -8 and -9 specific siRNAs. GAPDH and non-targeting siRNAs were used as controls. Western blot analysis showed that the amount of caspase-3, -4, -8, -9 and GAPDH was decreased by specific siRNAs (Figure 3C), and no effect was observed when cells were treated with the non-targeting siRNA control. The effect of the caspase-3, -4, -8, -9 or non-targeting knockdown control on viability of osajin-treated TW04 cells was also assessed (Figure 3D). Cell viability of 5 µM osajin-treated TW04 cells was increased by transfection with caspase-3, -4, -8 or -9 siRNA, respectively, compared with cells transfected with non-targeting siRNA control. Altogether, osajin-induced apoptosis in TW04 cells takes place mainly through the activation of caspase-3, -8 and -9, and is partly associated with the activation of caspase-4.

### Osajin induces loss of mitochondrial membrane potential and release of cytochrome *c* into the cytosol of TW04 cells

**Figure 4:**
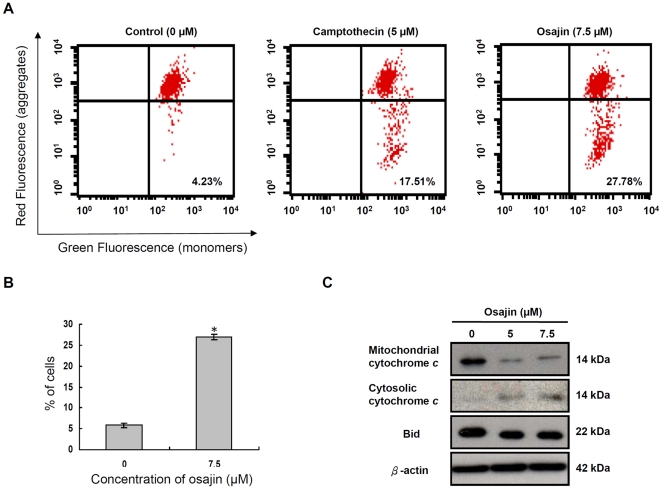
Osajin causes disruption of mitochondrial membrane potential and release of cytochrome *c* into the cytosol. (**A**) TW04 cells were treated with 7.5 µM osajin for 24 h before staining with MitoCapture™, a cationic dye. The mitochondrial membrane potential was measured by flow cytometry. The shift-down of fluorescence from red to green indicates the collapse of mitochondrial membrane potential. Camptothecin was used as a positive control for the disruption of mitochondrial membrane potential. The percentage of cells with disrupted mitochondrial membrane potential is indicated. (**B**) The data indicate the percentage of cells with disruption of mitochondrial membrane potential. Results are shown as means ± SE of four independent experiments (*P<0.05 versus 0 µM control). (**C**) Proteins prepared from cells treated with 5 µM or 7.5 µM osajin for 24 h were subjected to Western blot for measurement of Bid and cytochrome *c* in the cytosol. β-actin was used as internal control to ensure that equal amounts of proteins were loaded in each lane.

Activation of caspase-9 is mostly due to disruption of mitochondrial transmembrane potential and the release of cytochrome *c*. The osajin effect on mitochondrial membrane potential was studied by flow cytometry after staining with MitoCapture™, a cationic dye. Exposure of TW04 cells to osajin for 24 h caused a loss of red fluorescence (Figure 4A,B), indicating the disruption of mitochondrial transmembrane potential, which was comparable to the mitochondrial depolarization due to treatment with 5 µM camptothecin as the positive control.

The release of cytochrome *c* was next examined by Western blot analysis. Cytochrome *c* was hardly detectable in the cytosol of untreated NPC cells, but was increased following treatment with 5 µM and 7.5 µM osajin for 24 h, respectively (Figure 4C). Thus, osajin induced both loss of mitochondrial membrane potential and release of cytochrome *c* into the cytosol.

### Osajin induces FasL/CD95L expression in TW04 cells

**Figure 5:**
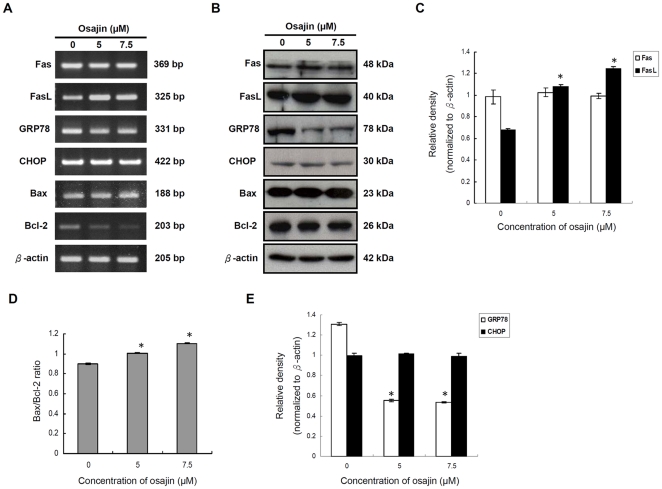
Effect of osajin on the expression of Fas/CD95, FasL/CD95L, GRP78/BiP, CHOP/GADD153 and Bcl-2 family proteins. TW04 cells were treated with various concentrations of osajin for 24 h. (**A**) RNA was isolated from cells treated with 5 µM or 7.5 µM osajin. Two µg of RNA was reversely transcribed into cDNA using oligo (dT) primers. RT-PCR analysis was performed using primers specific for Fas, FasL, GRP78, CHOP, Bax and Bcl-2 genes and also the internal control gene, β-actin. (**B**) Cell lysates were prepared for SDS-PAGE followed by Western blot for Fas, FasL, GRP78, CHOP, Bax and Bcl-2, with β-actin as a loading control. (**C**) to (**E**) Results are presented as the relative densities of protein bands normalized to β-actin. The data shown are the means ± SE of three individual experiments (*P<0.05 versus 0 µM control).

**Figure 6:**
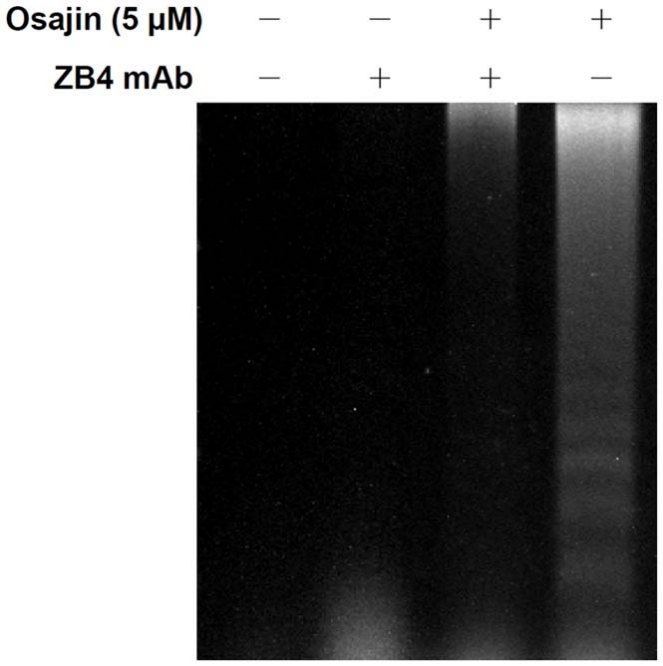
DNA fragmentation assay shows inhibition of apoptosis in osajin-treated NPC cells by the anti-Fas mAb ZB4. TW04 cells were preincubated for 2 h with 500 ng/ml ZB4 mAb, and osajin (5 µM) was then added and incubation was continued for 24 h prior to cell harvesting and agarose gel electrophoresis of fragmented DNA.

Activation of the initiator caspase, caspase-8, often results from stimulation of death receptors of the TNF receptor superfamily such as Fas/CD95 or TRAIL receptor [31]. To determine whether osajin affects levels of Fas and FasL in TW04 cells, the mRNA and protein levels of Fas and FasL in untreated cells and cells treated with 5 µM or 7.5 µM of osajin were evaluated. As shown in Figure 5, the expression level of FasL increased after exposure to osajin for 24 h, whereas Fas expression remains unchanged. To confirm that the Fas/FasL pathway involves osajin-induced apoptosis, we used the blocking anti-Fas mAb and determined whether it affected osajin-induced apoptosis. Figure 6 shows that osajin-induced apoptosis in TW04 cells was significantly attenuated by preincubation with anti-Fas mAb. This result suggests that osajin induces increased FasL expression in which contributes to apoptosis of NPC cells.

### Osajin modulates the level of Bcl-2 family proteins in TW04 cells

**Figure 7:**
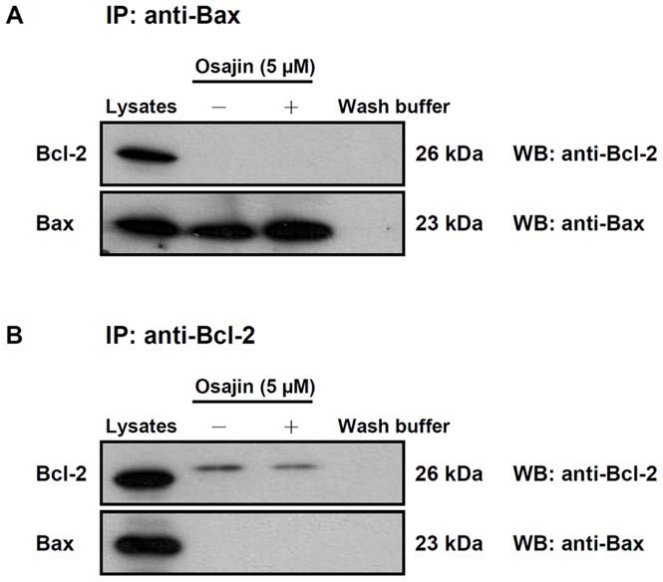
Co-immunoprecipitation of Bax and Bcl-2 in osajin-treated NPC cells. Immunoprecipitation assays were preformed as described in Materials and Methods. TW04 cell lysates served as a positive control; while immunoprecipitation wash buffer served as a negative control. Cell lysates of untreated and osajin-treated TW04 cells were immunoprecipitated by primary anti-Bax or anti-Bcl-2 antibodies. Western blots were preformed to demonstrate whether osajin treatment affected the heterodimerization of Bax and Bcl-2.

The release of cytochrome *c* is tightly regulated by members of the Bcl-2 family known to play an important role in regulation of apoptosis [32]. To examine whether expression of Bcl-2 or Bax may be modulated by osajin, TW04 cells were treated with 5 µM or 7.5 µM of osajin for 24 h, and mRNA and protein levels for Bcl-2 and Bax were examined. As shown in Figure 5A and B, osajin treatment significantly decreased the level of Bcl-2 mRNA, and caused a smaller but significant decrease in Bcl-2 protein. Concomitantly, the level of Bax proteins increased slightly, thereby causing a significant increase in the Bax/Bcl-2 ratio that favors apoptosis (Figure 5D). To further determine whether osajin inhibits the function of Bcl-2 by blocking heterodimerization of Bcl-2 and Bax, co-immunoprecipitation with Bcl-2 and Bax was performed. Direct interaction between Bcl-2 and Bax was not detected in either untreated or osajin-treated TW04 cells (Figure 7). In addition, the amount of full-length Bid was reduced by 20–30% in the cytosolic fraction of 5 and 7.5 µM osajin-treated NPC cells (Figure 4C).

### Differential regulation of GRP78/BiP and CHOP/GADD153 in osajin-treated TW04 cells

Under conditions associated with ER stress, GRP78/BiP is recruited to misfolded proteins to facilitate their proper folding, thus serving as a major player in the survival program [33]. On the other hand, CHOP/GADD153 is one of several molecules mediating ER stress-induced apoptosis [28]. Thus, the effect of osajin on mRNA expression and protein production levels of GRP78 and CHOP was analyzed in TW04 cells. Treatment with osajin in TW04 cells dramatically down-regulated the levels of GRP78 in a dose-dependent manner. In contrast, the level of CHOP was unchanged in cells treated with different concentrations of osajin (Figure 5E).

## Discussion

Chemoprevention by the use of naturally-occurring dietary substances is attracting mounting attention as a practical approach to reduce the ever-increasing incidence of cancers [34]. Currently some promising dietary chemopreventive compounds include (-)-epigallocatechin gallate (EGCG), resveratrol, lupeol, delphinidin, curcumin, genistein, isothiocyanates, lycopene and other substances which have in common their ability to induce apoptosis of cancer cells [35]. Although previous studies have suggested that osajin could be used in cancer prevention [8], the effects of osajin on human NPC cells have not been previously characterized. Results from this study show that osajin is cytotoxic for three different types of NPC cell lines. Using the type 3 NPC TW04 cell line as the study model, the mechanism of cytotoxicity was found to depend on induction of apoptosis. Our results are thus consistent with previous studies.

Results from this study indicate that caspase-3, -4, -8 and -9 are all involved in osajin-induced apoptosis of TW04 cells. Although there seems to be no dominant pathway revealed by the use of caspase inhibitors and gene knockdown assays, a significant increase of caspase-3 activity is due to synergistic effects of caspase-4, -8 and -9. Furthermore, as none of the caspase inhibitors or siRNAs rescued the NPC cells completely, our results suggested that the caspases induced apoptosis through nonredundant pathways. However, we can not exclude the possibility that other mechanisms may regulate cell survival and apoptosis in osajin-treated NPC cells. NPC cell treatment with osajin results in the disruption of mitochondrial transmembrane potential, release of cytochrome *c* from mitochondria, and activation of caspases-9 and -3.

The apoptotic pathway is also regulated by members of the Bcl-2 family of proteins. This family includes a number of proteins with homologous amino acid sequences, including antiapoptotic members such as Bcl-2 and Bcl-x_L_, as well as proapoptotic members including Bax and Bak [36], [37]. Overexpression of Bax promotes cell death [38]. Conversely, overexpression of Bcl-2 stimulates formation of a Bcl-2-Bax heterodimer, thereby neutralizing the proapoptotic activity of Bax [39]. Results from this study show that osajin-induced apoptosis in human NPC cells is accompanied by down-regulation of Bcl-2 and up-regulation of Bax. Although Bcl-2 has been reported to bind to Bax, no interaction was detectable in untreated and osajin-treated TW04 cells. The undetectable Bcl-2/Bax interaction is not surprising, because the Bcl-2 protein can bind to a variety of non-Bcl-2 family proteins, such as p53, Raf-1 and Ras [40]. Previous studies also showed that antiapoptotic Bcl-2 proteins interact with Bax weakly [41]. Moreover, the protein level of Bcl-2 is much lower than that of Bax in TW04 cells which could explain why the Bcl-2/Bax interaction was not detected in TW04 cells. However, down-regulation of Bcl-2 and up-regulation of Bax may contribute to the alteration of the permeability of mitochondria, release of cytochrome *c* into the cytosol, activation of caspase-3, and ultimately cell death.

The Fas pathway is involved in osajin-induced apoptosis of NPC cells. Treatment with osajin triggered the increased expression of FasL which can stimulate the Fas receptor in an autocrine or paracrine manner. Besides the membrane-bound form, soluble FasL (sFasL) may also be obtained by cleavage of membrane-bound FasL (mFasL) through a metalloprotease-like enzyme [42]. Both mFasL and sFasL bind to Fas and subsequently trigger apoptosis through the Fas/FasL system, but sFasL has been reported to be a weaker inducer of apoptosis than mFasL [43]. Osajin activates expression of mFasL, which usually leads to activation of caspase-8. As we did not measure the effect of osajin on the amount of sFasL produced, we can not rule out the possibility that osajin may also affect the amount of sFasL. Interestingly, osajin did not affect expression of Fas, suggesting that expression of Fas and mFasL may be under different regulatory pathways. Furthermore, caspase-8 is most likely activated by the increased expression of FasL, rather than through enhanced sensitivity of the Fas pathway in osajin-treated cells.

The activation of the death receptor pathway by osajin also connects to the mitochondrial pathway, which involves cleavage of the Bcl-2 family member Bid by caspase-8. Our data show a 20–30% decrease of expression of the full-length Bid protein in the cytosolic fraction of NPC-TW04 cells after treatment with osajin. However, no cleaved Bid was detected by Western blot analysis after three independent experiments. Previous studies have shown that tBid is rapidly degraded by the ubiquitin proteolytic system, and inhibition of the proteasome therefore increases apoptosis by increasing tBid levels [44]. It may be possible that tBid is already degraded after osajin treatment under our experimental conditions.

ER stress is also involved in apoptosis triggered by osajin treatment. ER stress leads to proteolytic cleavage of caspase-12 in the mouse and caspase-4 in humans, but both caspases localize to the cytoplasmic side of the ER membrane [26]. Furthermore, Kim *et al.* found that neuronal apoptosis in infantile neuronal ceroid lipofuscinosis is caused by ER stress-mediated caspase-4 activation, leading to subsequent caspase-3 activation and apoptosis [45]. Our results showed that osajin activates caspase-4 and caspase-3. Another protein, CHOP, known as GADD153 and a transcription factor that decreases the expression of Bcl-2, can also induce apoptosis [30]. CHOP/GADD153 is present at low levels under normal conditions, but is robustly expressed in response to ER stress [46]. Besides CHOP, the ER chaperone GRP78/BiP, which shows antiapoptotic properties and whose expression is widely reported in human cancer, is a central regulator of ER homeostasis [25]. Using a genetic model of breast cancer in *GRP78*–deficient mice, *GRP78* heterozygous mice have no effect on organ development or antibody production but show significant inhibition of *in situ-*generated tumor growth and tumor angiogenesis [47]. Recent studies have also revealed that GRP78 can directly inhibit ER stress-induced apoptosis through direct binding and subsequent inhibition of caspase-7 activation [48]. In this study, treatment of osajin down-regulated GRP78 expression in NPC cells. However, the level of CHOP protein was constant in osajin-treated NPC cells. Thus, osajin may modulate ER stress-mediated apoptosis of NPC cells through regulation of GRP78.

**Figure 8:**
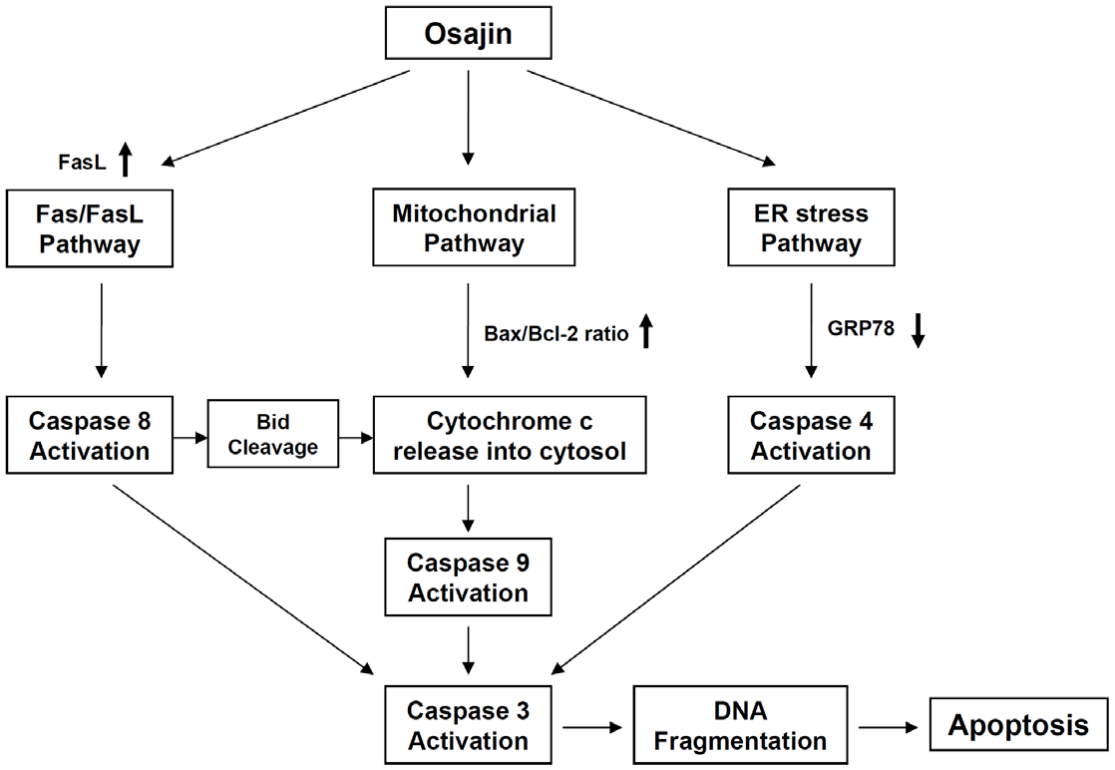
Schematic model for the mechanisms of osajin-induced apoptosis in human NPC cells. Osajin induces apoptosis in human NPC cells through multiple apoptotic pathways, including death receptor-, mitochondria- and ER stress-dependent pathways.

In conclusion, although we have not identified yet a causal link between the different apoptotic pathways and the target molecules of osajin remain unknown, our results indicate that osajin triggers apoptosis of human NPC cells through multiple apoptotic pathways (Figure 8). This is achieved by activating different caspases via three pathways: surface death receptors, mitochondria, and ER stress. These findings suggest that osajin should be considered as a potential broad-range chemopreventive agent for NPC.

## Materials and Methods

### Reagents

Osajin was purchased from Bode United Technology (Beijing, China). Dimethyl sulfoxide (DMSO), bovine serum albumin (BSA) and protease inhibitor cocktail were obtained from Sigma (St Louis, MO, USA). Z-DEVD-FMK (caspase-3 inhibitor), Z-LEVD-FMK (caspase-4 inhibitor), Z-IETD-FMK (caspase-8 inhibitor), and Z-LEHD-FMK (caspase-9 inhibitor) were purchased from BioVision (Mountain View, CA, USA). Osajin was dissolved in DMSO to make a stock solution of 50 mM stored at −20°C and diluted to the desired concentration before use. Cell culture medium (DMEM, F-12), fetal bovine serum (FBS), penicillin, streptomycin, and trypsin-EDTA were purchased from Gibco BRL (Grand Island, NY, USA).

### Cell lines and cell culture

Three different types of human NPC cell lines were studied: TW076 (type 1 NPC, squamous cell carcinoma, EBV negative) [49], CG1 (type 2 NPC, non-keratinizing carcinoma, EBV positive) [50], and TW04 (type 3 NPC, undifferentiated carcinoma, EBV negative) [51]. TW04 and TW076 cells were cultured in DMEM, and CG-1 cells were cultured in DMEM/F-12 (3∶1, v/v) supplemented with 10% (v/v) FBS, 100 units/ml penicillin and 100 µg/ml streptomycin at 37°C in a 5% CO_2_ incubator with saturated humidity.

### Cell viability assay

Cell viability was determined using the MTT-based in vitro toxicology assay kit (Sigma), which measures colorimetrically a purple formazan compound produced by viable cells. Briefly, NPC cells were seeded in 96-well plates (5×10^3^/well) and allowed to adhere for 24 h. The medium was then substituted by a fresh one containing different concentrations (0.01–10 µM) of osajin for another 24 h. For the time-course assay, the incubation times with 5 µM of osajin were 6, 12, 24, 36 and 48 h. After incubation, 10 µl of 5 mg/ml of MTT was added to each well and incubated for 4 h at 37°C. Elution of the precipitate was performed with 100 µl of solubilization solution. Cell viability was calculated from absorption values obtained at 570 nm using an automated ELISA reader (Cape Code, UK). All experimental concentrations were performed in triplicate.

### Annexin V-PI binding assay

To measure apoptotic cell death, an Annexin V-FITC Apoptosis Detection Kit (BioVision) together with flow cytometry was used according to the manufacturer's protocol in order to quantify the externalization of phosphatidylserine by flow cytometry. In brief, 5×10^5^ cells grown to 60% confluence were treated with 0, 5 and 7.5 µM of osajin for 24 h. For the Annexin V/PI binding assay, both floating and adherent cells were collected and washed with serum-containing media. Collected cells were washed with serum-containing media before being resuspended in 500 µl of 1× binding buffer, followed by addition of 5 µl of Annexin V-FITC and 5 µl of PI to the cell suspension. The mixture was then incubated for 5 min at room temperature in the dark and immediately analyzed with the flow cytometer (Becton Dickinson, San Jose, CA, USA) and Cell Quest 3.3 software (Becton Dickinson).

### Detection of DNA fragmentation

Agarose gel electrophoresis of fragmented DNA was preformed as previously described [52]. DNA was prepared from untreated and osajin-treated cells by standard digestion and organic extraction. Extracted DNA was separated by electrophoresis in 1.2% agarose gel and visualized with ethidium bromide staining. For blocking experiments [53], TW04 cells were preincubated with blocking 500 ng/ml anti-Fas mAb ZB4 (Millipore, Billerica, MA, USA) for 2 h, and osajin (5 µM) was subsequently added and incubation continued for 24 h prior to harvesting of cells. In some experiments, apoptosis was also evaluated by assessing the enrichment of nucleosomes in the cytoplasm, compared with control cells. Cytoplasmic DNA fragments were quantified with a cell death detection ELISA^PLUS^ kit (Roche, Mannheim, Germany). Briefly, cells were plated in triplicate in a 96-well plate at a density of 1×10^4^ cells per well and were treated with osajin at varying concentrations (1–10 µM) for 24 h. The level of apoptosis was expressed as the enrichment factor = absorbance of the sample/absorbance of the corresponding control.

### Caspase activity assay

The activity of caspase-3, -4, -8 and -9 was measured using a colorimetric assay kit (BioVision) according to the manufacturer's instructions. Cells were incubated for 24 h with different concentrations of osajin. The cell lysate from 1–5×10^6^ cells was incubated at 37°C for 2 h with 200 µM DEVD-*p*-nitroanilide (*p*NA) (caspase-3 substrate), LEVD-*p*NA (caspase-4 substrate), IETD-*p*NA (caspase-8 substrate), or LEHD-*p*NA (caspase-9 substrate). Spectrophotometric detection of the chromophore *p*NA after cleavage from the labeled caspase substrates was then performed. Samples were read at 405 nm and the enzyme activity was expressed as fold-increase over untreated control samples.

### Transfection of small interfering RNA (siRNA)

TW04 cells were seeded in antibiotic-free media for 24 h prior to transfection with siRNA targeting caspase-3, caspase-4, caspase-8, caspase-9 (ON-TARGETplus SMARTpool), corresponding ON-TARGETplus GAPD control pool and non-targeting control pool, which were purchased from Dharmacon (Thermo Fisher Scientific, Lafayette, CO, USA). Cells were transfected with 25 nM siRNA in antibiotic-free complete media using DharmaFECT I Reagent (Dharmacon) according to the manufacturer's protocol. Forty-eight hours post-transfection, cells were harvested and assayed for protein expression levels of the target of interest.

### Mitochondrial membrane potential detection assay

The MitoCapture™ Mitochondrial Apoptosis Detection kit (BioVision) for detection of mitochondrial transmembrane transition events in live cells was used. Growing cells (60% confluence) were treated with 7.5 µM of osajin for 24 h. Collected cells were suspended in 1 ml of the diluted MitoCapture solution, followed by incubation at 37°C for 20 min. After centrifugation, the cell pellet was resuspended in 1 ml of the pre-warmed incubation buffer before measurement by flow cytometry.

### Western blot analysis

Twenty-four hours after osajin treatment, cells were washed twice with cold PBS before incubation in ice-cold lysis buffer containing 150 mM NaCl, 50 mM Tris-HCl (pH 7.5), 0.5% Nonidet P-40, 1 mM PMSF, 1 mM NaF, 1 mM sodium orthovanadate, 1 mM DTT, 10 mM β-glycerophosphate, and 4 µg/µl complete protease inhibitor cocktail over ice for 30 min. Cell suspensions were then centrifuged for 30 min at 15,000× *g* at 4°C, and the supernatant (total cell lysates) was collected and stored at −70°C. Cytosolic and mitochondrial extracts were prepared using a Mitochondrial/Cytosol Fractionation kit (BioVision). The protein concentration was determined using Bradford assay (Bio-Rad, Richmond, CA, USA). Equal amounts of protein were separated by electrophoresis in 10 to 15% SDS-polyacrylamide gels. Protein samples were transferred onto PVDF membranes (Millipore), and detected by the appropriate primary and secondary antibodies before visualization using an enhanced chemiluminescence detection kit (Millipore). The antibodies anti-Bid, anti-caspase-3, anti-caspase-8, anti-caspase-9 and anti-cytochrome *c* were from Cell Signaling Technology (Beverly, MA, USA); anti-Bax, anti-Bcl-2, anti-CHOP, anti-Fas, anti-FasL, anti-GAPDH and anti-GRP78 antibodies were from Santa Cruz Biotechnology (Santa Cruz, CA, USA); anti-caspase-4 was from MBL (Naka-ku, Nagoya, Japan); and anti-β-actin was from Novus Biologicals (Littleton, CO, USA). The secondary antibodies were horseradish peroxidase-conjugated anti-rabbit and anti-mouse IgG (Santa Cruz Biotechnology).

### Immunoprecipitation

The immunoprecipitations were performed according to the Catch and Release v2.0 Kit (Millipore) following the kit recommendations. Cell lysates were incubated with anti-Bax or anti-Bcl-2 antibodies at 4°C overnight on a rotator by using Catch and Release spin columns. The column was washed 3 times with 1× Wash Buffer, spinning at 2,000× *g* 15–30 seconds for each wash. Proteins bound to the beads were eluted by denaturing elution buffer, separated by SDS-PAGE and immunoblotted with antibodies against proapoptotic and antiapoptotic proteins to identify the binding of Bcl-2 family proteins.

### RNA isolation and reverse transcriptase-polymerase chain reaction (RT-PCR)

Total RNA from TW04 cells was extracted with the TRIzol reagent, according to the manufacturer's instructions (Invitrogen, Carlsbad, CA, USA). Two micrograms of RNA were reversely transcribed in 20 µl reaction volumes containing an oligo (dT) primer (Invitrogen) and the M-MLV reverse transcriptase (Promega, Madison, WI, USA). The cDNA for Bax, Bcl-2, CHOP, GRP78, Fas, FasL and β-actin were amplified by PCR with specific primers: Bax forward primer 5′-TCTGACGGCAACTTCAACTG-3′, and reverse primer 5′-TTGAGGAGTCTCACCCAACC-3′; Bcl-2 forward primer 5′-TCCATGTCTTTGGACAACCA-3′, and reverse primer 5′-CTCCACCAGTGTTCCCATCT-3′; CHOP forward primer 5′-GCACCTCCCAGAGCCCTCACTCTCC-3′, and reverse primer 5′-GTCTACTCCAAGCCTTCCCCCTGCG-3′; GRP78 forward primer 5′-GCTCTCGAATTCCAAAG-3′, and reverse primer 5′-TTTGTCAGGGGTCTTTCACC-3′; Fas forward primer 5′-GGATGAACCAGACTGCGTG-3′, and reverse primer 5′-CTGCATGTTTTCTGTACTTCC-3′; FasL forward primer 5′-CTCTGGAATGGGAAGACACC-3′, and reverse primer 5′-ACCAGAGAGAGCTCAGATACG-3′; β-actin forward primer 5′-GAGACCTTCAACACCCCAGCC-3′, and reverse primer 5′-GGATCTTCATGAGGTAGTCAG-3′. The PCR products from the same sample were electrophoresed in a 2% agarose gel and visualized by ethidium bromide staining on an image system.

### Statistical analysis

Data were expressed as mean ± SE. Comparisons between untreated control cells and osajin-treated cells were made using Student's *t*-test, unless otherwise stated. The statistically significant levels were set at *P<0.05.
